# Construction of pomelo peel essential oil pickering emulsion and its impact on the freeze-thaw stability of surimi gel

**DOI:** 10.1515/biol-2025-1301

**Published:** 2026-03-16

**Authors:** Hao Song, Lin Zhao, Hu Zhang, Baoming Tian

**Affiliations:** School of Food and Health, Zhejiang Institute of Economics and Trade, No. 280 Xuelin Road, Hangzhou City, Zhejiang Province, 310018, China; College of Food Science and Technology, Zhejiang University of Technology, No. 18 Chaowang Road, Hangzhou City, Zhejiang Province, 310014, China

**Keywords:** freeze-thaw (F-T) cycles, *β*-cyclodextrin (β-CD) stabilization, optimization, water-holding capacity (WHC), low-fat

## Abstract

Surimi gel products, often subjected to multiple freeze-thaw (F-T) cycles during storage and transport, suffer from texture deterioration and drip loss. High-fat additives, used to improve mouthfeel and gel properties, raise health concerns. This study aimed to improve F-T stability and reduce fat content by constructing a *β*-cyclodextrin (β-CD) stabilized Pickering emulsion using pomelo peel essential oil (PPEO). Optimal conditions were 3 % *β*-CD, pH 7.5, and an oil-to-water ratio of 2:8, yielding an emulsion with a particle size of 74.19 nm and 97.73 % emulsification activity. Replacing 0–100 % of basa fish oil with this emulsion in surimi gel significantly inhibited hardness loss after three F-T cycles (*p* < 0.05). The 50 % substitution group promoted a dense gel network, achieving the highest whiteness and a water-holding capacity (WHC) of 83.25 % post F-T. Substituting 50 % of fat with PPEO Pickering emulsion enhanced F-T stability, antioxidant properties, whiteness, and WHC through microstructural optimization, offering a novel strategy for low-fat surimi product development.

## 1 Introduction

Surimi gel is a fish-derived product prepared by mechanically chopping and heat-treating a mixture of surimi, lipids, water, salt, and other ingredients. Valued for high nutritional value and convenience, these products require long-term frozen storage. However, temperature fluctuations during storage and transportation subject them to multiple freeze-thaw (F-T) cycles. The repeated formation and melting of ice crystals damage muscle tissue, leading to texture changes, drip loss, and compromised quality [1], 2]. Furthermore, traditional processing often adds high-fat pork back fat to maintain mouthfeel, gel strength, elasticity, water-holding capacity (WHC), and oiliness [3]. Excessive consumption of high-fat meat products is linked to obesity, cardiovascular disease, diabetes, and cancer [4]. Therefore, minimizing quality deterioration from F-T cycles while reducing fat content in surimi gels is crucial.

Commercial cryoprotectants like phosphates, sucrose, and sorbitol can enhance F-T stability but may have adverse health effects when consumed excessively [5]. Alternative strategies using low-sweetness, low-calorie agents have been explored. For instance, soluble soybean polysaccharide combined with liquid nitrogen freezing reduced ice crystal formation in surimi but is costly [5]. Membrane-separated hydrolysates of silver carp showed cryoprotective activity [6]. Soybean lecithin liposomes with trehalose improved WHC but can interfere with protein-protein interactions, reducing gel strength [7]. Feruloyl oligosaccharides from rice bran improved WHC but not whiteness [8]. Thus, conventional approaches have not fully resolved F-T induced quality deterioration.

Pickering emulsions, stabilized by micro/nano solid particles, offer superior stability, lower emulsifier requirements, and environmental friendliness compared to surfactant-stabilized emulsions. Due to their high oil content and favorable rheology, they are used as fat replacers in various foods [9], [10], [11]. Feng et al. [12] found that quinoa protein-stabilized Pickering emulsion reduced free water and drip loss in surimi gels after F-T cycles. However, protein-stabilized emulsions can be sensitive and unstable [13]. Zhang et al. [14] used a cinnamon bark essential oil Pickering emulsion for shrimp preservation, improving WHC and sensory indices. This highlights the potential of plant essential oil-based Pickering emulsions in aquatic product preservation.

Pomelo peel essential oil (PPEO), extracted from *Citrus grandis* L. peel, is rich in unsaturated fatty acids and possesses antioxidant and antimicrobial activities. A stable PPEO Pickering emulsion could reduce saturated fat in surimi products while improving F-T stability [15]. Current research on plant essential oil Pickering emulsions in meat products focuses on coating methods, with limited studies on processed aquatic products [16], 17]. This study aimed to prepare a *β*-cyclodextrin (β-CD) stabilized PPEO Pickering emulsion, use it to replace fat at different levels in surimi gel, and investigate its effects on quality during F-T cycles, providing a theoretical foundation for application in frozen minced meat products.

## 2 Materials and methods

### 2.1 Materials

Frozen basa fish fillets were supplied by Shanghai Beite Food Co. (Shanghai, China) and stored at −18 °C. Basa fish oil was sourced from Asian Fish Oil (Tianjin) Biotechnology Co. (Tianjin, China). PPEO was obtained from Shanghai Yuanye Biotechnology Co. (Shanghai, China). *β*-CD was provided by Shanghai Macklin Biochemical Technology Co. (Shanghai, China). Corn starch was supplied by Shanghai Fengwei Industrial Co. (Shanghai, China). All other reagents were analytical grade.

### 2.2 PPEO pickering emulsion preparation

The emulsion was prepared following Cen et al. [18] with modifications. A precise amount of *β*-CD was dissolved in 30 mL water in a 60 °C water bath. The solution pH was adjusted using a portable pH meter (PHSJ-4F, Wuhan Tianyili Instrument Equipment Co., Ltd., China) and 1.0 mol/L NaOH or HCl. A predetermined amount of PPEO was added to achieve the preset oil-to-water ratio. The mixture was emulsified using a high-speed homogenizer (FJ200-SH, Shanghai Huxi Industrial Co., Ltd., China) at 10,000 r/min for 20 s per cycle, with 10 s intervals, repeated 6 times.

#### 2.2.1 Effect of *β*-CD concentration

With an oil-to-water ratio of 1:3 and pH 7.5, the effects of *β*-CD concentrations (1 %, 2 %, 3 %, 4 %, 5 %) on average particle size and emulsification stability index (ESI) were investigated.

#### 2.2.2 Effect of emulsion pH

Using an oil-to-water ratio of 1:3 and 3 % *β*-CD, the effect of pH (7.0, 7.5, 8.0, 8.5, 9.0) on particle size and ESI was studied.

#### 2.2.3 Effect of oil-to-water ratio

With 3 % *β*-CD and pH 7.5, the effects of oil-to-water ratios (4:6 (*φ* = 60 %), 7:13 (*φ* = 65 %), 3:7 (*φ* = 70 %), 1:3 (*φ* = 75 %), 2:8 (*φ* = 80 %)) on particle size and ESI were examined.

#### 2.2.4 Orthogonal experimental design

An L_9_ (3^4^) orthogonal experiment was conducted based on single-factor tests. Independent variables were *β*-CD concentration, pH, and oil-to-water ratio; dependent variables were average particle size and ESI. Particle size directly reflects emulsification efficiency and the compactness of the interfacial particle layer, with smaller sizes indicating greater interfacial area and stability. ESI measures the emulsion’s ability to resist phase separation during storage, representing its practical stability. Optimizing both parameters ensures the production of an emulsion with favorable formation and storage properties, essential for its integration into surimi gel. Factor levels are shown in Table 1.

**Table 1:** Factor levels of the orthogonal experiment.

Level	Factor		
	A, *β*-CD concentration (%)	B, emulsion pH value	C, oil-to-water ratio (v/v)
1	2.5	7.0	3:7
2	3.0	7.5	1:3
3	3.5	8.0	2:8

#### 2.2.5 Determination of average particle size

A 0.5 mL emulsion aliquot was diluted 10-fold with double-distilled water and analyzed using a laser particle size analyzer (Bettersize 2000, Dandong Bettersize Instruments Ltd., China). Measurements were performed in triplicate.

#### 2.2.6 Determination of ESI

The freshly prepared emulsion was stored at room temperature for 24 h. The total mixture volume (V, mL) and emulsified phase volume (v, mL) were measured. The ESI (%) was calculated using equation (1).

(1) $$\text{ESI }\left(\%\right)=\text{Volume of emulsified phase }/\text{ Volume of mixture}$$

#### 2.2.7 Calculation of comprehensive score

The method described by Souza et al. [19] was adapted with modifications. The particle size and ESI data were normalized for comprehensive evaluation. Particle size data were transformed using equation (2), and both indices were standardized to obtain a normalized score Z using equation (3). A weighted comprehensive score F was then calculated using equation (4).

The weights of the indices (sum to 1, expressed as *X* = {*x*_1_, *x*_2_}) were determined by 10 evaluators using the user survey method and binary comparison method, where the more important index was scored 1, the less important scored 0, and self-comparison scored 1.

(2) $$Y_{1}^{\prime }=\max\left(Y_{1}\right)-Y_{1}$$

(3) $$Z=\left(Y_{1}^{\prime }-\min\left(Y_{1}^{\prime }\right)\right)/\left(\max\left(Y_{1}^{\prime }\right)-\min\left(Y_{1}^{\prime }\right)\right)\;\left(i=1,2\right)$$

(4) $$F=x_{1}Z_{1}+x_{2}Z_{2}$$

where: $Y_{1}^{\prime }$ is transformed particle size data; *Y*_1_ is original particle size data; *Z* is normalized index score; min ( $Y_{1}^{\prime }$ ) and max ( $Y_{1}^{\prime }$ ) represent minimum and maximum values of the transformed indices; *F* is comprehensive score; *x*_1_ and *x*_2_ represent weights of particle size and ESI in the comprehensive evaluation.

### 2.3 Preparation of surimi gel

The surimi gel was prepared according to Sun et al. [20] with slight modifications. Briefly, frozen basa fish fillets were thawed, trimmed, and chopped without additives for 2 min, followed by chopping with 3 % salt for 5 min. Basa fish oil and/or PPEO Pickering emulsion (*φ* = 75 %), along with two-thirds of the ice water, were then added and chopped for 5 min. The total mass of the oil phase (from fish oil and/or emulsion) and ice water was maintained at 20 % of the surimi mass. To ensure consistent total moisture and oil phase content across all treatment groups when substituting fish oil with the emulsion, the amount of externally added ice water was adjusted downward proportionally, accounting for the water already present in the emulsion (which had an oil phase volume fraction *φ* of 75 %). Detailed addition amounts for each substitution level are provided in Table 2 (0 % replacement served as the control). Subsequently, 10 % corn starch and the remaining one-third of the ice water were added, followed by chopping for an additional 5 min. The mixture was manually formed into surimi gels (approximately 20 g each), cooked at 70–80 °C until fully set, and then cooled.

**Table 2:** Addition amounts for different PPEO Pickering emulsion substitution levels.

Emulsion substitution ratio (%)	Basa fish oil (%)	Emulsion (*φ* = 75 %) (%)	Ice water (%)
0	10.00	0.00	10.00
25	7.50	3.33	9.17
50	5.00	6.67	8.33
75	2.50	10.71	6.79
100	0.00	13.33	6.67

### 2.4 F-T cycles

Following Zhang et al. [21] with modifications, surimi gels were frozen at −18 °C for 5 days, then thawed at 4 °C for 12 h. This constituted one cycle, repeated three times.

### 2.5 Texture profile analysis (TPA)

Gels were cut into cylinders (15 mm height). Texture was analyzed using a TA-XT Plus texture analyzer with a P/75 probe (Stable Micro Systems Ltd., UK). Parameters: 30 % compression, pre-test speed 2.0 mm/s, test speed 1.0 mm/s, post-test speed 2.0 mm/s, trigger force 5 g. At least four replicates were measured per sample. Outliers were identified and excluded before calculating the average value. Specifically, an outlier was defined as any datum for hardness or chewiness whose absolute deviation from the mean of the remaining replicates exceeded three times the standard deviation. The average value was then calculated from the remaining replicates.

### 2.6 Determination of antioxidant capacity

Thiobarbituric acid reactive substances (TBARS) value was determined following Li et al. [22] with modifications. A 5 g sample was dispersed in 50 mL of 0.1 % EDTA and 7.5 % trichloroacetic acid, homogenized at 12,000 r/min for 2 min, and filtered. Filtrate (10 mL) was mixed with 10 mL of 0.02 mol/L TBA solution, heated in boiling water for 40 min, cooled, mixed with 5 mL chloroform, and centrifuged at 3,000 r/min for 10 min. The TBARS value was calculated using equation (5):

(5) $$\text{TBARS }\left(\text{mg}/\text{kg}\right)=\frac{\;A532‐A600}{155\times m}\times 72.06\times 1000$$

where: A532 and A600 respresent absorbance at 532 nm and 600 nm,; 155 is molar absorptivity; m is sample mass (g); 72.06 is molar mass of malondialdehyde (g/mol).

### 2.7 Colorimetric analysis

Following You et al. [23] with modifications, gels were cut into cylinders (15 mm high). Color indices (*L*, *a*, *b*) were measured using a colorimeter (CR-10, Konica Minolta Sensing, Japan). Whiteness (*W*) was calculated using equation (6):

(6) $$W=100-\sqrt{{\left(100‐L\right)\;}^{2}+a^{2}+b^{2}}$$

where: *W* is whiteness; *L* is lightness; *a*, *b* respresent chromaticity values (directly read from the colorimeter).

### 2.8 Determination of WHC

Following Zhang et al. [24] with modifications, approximately 3 g of sample was wrapped in filter paper and centrifuged at 10,000 r/min at 4 °C for 10 min. The sample mass was weighed before and after centrifugation, and WHC was calculated using equation (7):


(7) $$\text{WHC }\left(\%\right)=\text{Mass of the sample before centrifugation }/\text{ Mass of the sample after centrifugation}$$

### 2.9 Microstructure of surimi gel

Following Zhu et al. [25] with modifications, gel samples were cut into 1.0 × 1.0 × 0.5 cm pieces, fixed in 2.5 % glutaraldehyde at 4 °C overnight, rinsed with PBS buffer (pH 7.4), dehydrated through a graded ethanol series (30 %, 50 %, 70 %, 90 %, absolute ethanol), freeze-dried, sputter-coated with gold, and observed under a scanning electron microscope (Sigma 360, Carl Zeiss AG, Germany).

### 2.10 Statistical analysis

All measurements were performed in quadruplicate. Results are expressed as mean ± standard deviation. Graphs were generated using GraphPad Prism 9 (GraphPad Software, San Diego, CA, USA). Response surface analysis used Design-Expert 8.0.6 (Stat-Ease Inc., USA). Significant differences were analyzed by Duncan’s test using SPSS 20.0 (IBM Corp., USA). Principal component analysis was also conducted using SPSS. Differences were considered significant at *p* < 0.05.

## 3 Results and discussion

### 3.1 Single-factor experiments

#### 3.1.1 *β*-CD addition

**Figure 1:**
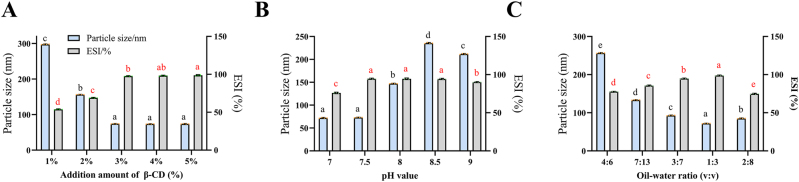
Effect of *β*-CD addition (**A**), pH (**B**), and oil-to-water ratio (**C**) on the particle size and ESI of pickering emulsions. The above values are expressed as mean ± SD (*n* = 4). Different letters indicate significant differences (*p* < 0.05) by Duncan’s multiple range test.

The amount of *β*-CD added significantly impacted the storage stability of the emulsion (Figure 1A). Increasing the *β*-CD concentration from 1 % to 3 % significantly reduced the emulsion particle size and increased the ESI. However, at concentrations of 4 % or above, changes in particle size and ESI were not significant. These results suggest that increasing *β*-CD concentration improves emulsion stability, which may be attributed to the adsorption of a greater number of cyclodextrin inclusion complex particles at the oil-water interface [26], 27]. These particles facilitate the formation of a sufficient interfacial film during emulsification and enhance its compactness, thereby reducing the average particle size and stabilizing the emulsion. When the *β*-CD concentration exceeded 3 %, the formation and reinforcement of the interfacial film reached saturation, resulting in stabilized particle size.

#### 3.1.2 pH value

The pH of the system influenced emulsion stability by altering the charge characteristics of the droplets [28]. As shown in Figure 1B, the average particle size of the emulsion changed significantly with pH. Relatively small particle sizes were observed at both pH 7.0 and pH 7.5, with no significant difference between them. Correspondingly, the ESI first increased and then decreased with increasing pH, reaching a maximum also at pH 7.5. This optimal stability at pH 7.5 may be attributed to the increased charge on the cyclodextrin-based interfacial film under slightly alkaline conditions. The enhanced electrostatic repulsion between oil droplets likely contributed to the improved stability, which is consistent with the observed minimum particle size [29].

#### 3.1.3 Oil-to-water ratio

The oil-to-water ratio significantly influenced the properties of the *β*-CD stabilized Pickering emulsion (Figure 1C). Particle size analysis revealed that the average droplet size varied with the oil phase proportion, reaching a minimum at a ratio of 1:3, followed by 2:8. This suggests a strong emulsifying capacity for the oil phase but a comparatively limited ability to incorporate the water phase [30]. Concurrently, the ESI increased significantly (*p* < 0.05) within the oil phase range of 60–75 %, peaking at the 1:3 ratio. The consistency between the minimal particle size and maximal ESI at this ratio indicates that *β*-CD particles form a tightly packed, robust interfacial network, thereby conferring optimal emulsion stability.

### 3.2 Orthogonal experiment results

Based on the single-factor experiments, an orthogonal experimental design was employed to optimize the PPEO Pickering emulsion preparation, with emulsion particle size and ESI as the dependent variables. The weights assigned to these variables (0.6 for ESI and 0.4 for particle size, as shown in Table 3) were determined by 10 evaluators using a combination of user survey and binary comparison methods. Considering the emulsion’s intended application in F-T cycles and as a fat replacer, the evaluators prioritized long-term storage stability (ESI) over minor initial differences in particle size for maintaining quality during frozen storage and distribution. To enable a combined assessment, the orthogonal experimental results for particle size and ESI were normalized to eliminate scale differences. A comprehensive score was then calculated using the weightings in Table 3 and used as the analytical outcome of the orthogonal experiment (Table 4).

**Table 3:** Weight assignment table of each factor.

Evaluation index	Scores of each factor			Weight
	Particle size	ESI	Total	
Particle size	10	2	12	0.4
ESI	8	10	18	0.6

**Table 4:** Orthogonal experiment results.

Experiment no.	Factor			Y_1_, particle size (nm)	Y_2_, ESI (%)	F, comprehensive score
	A, *β*-CD addition (%)	B, pH value	C, oil-to-water ratio (v/v)			
1	1	1	1	111.15	75.43	0.12
2	1	2	2	98.15	74.17	0.19
3	1	3	3	90.87	70.22	0.19
4	2	1	2	86.45	82.55	0.83
5	2	2	3	94.77	88.49	1.00
6	2	3	1	105.17	91.65	0.88
7	3	1	3	120.25	90.35	0.56
8	3	2	1	117.26	93.67	0.65
9	3	3	2	103.74	92.30	0.57
K_1_	0.17	0.50	0.55			
K_2_	0.90	0.61	0.53			
K_3_	0.59	0.55	0.58			
R	0.73	0.11	0.05			

Range analysis of the orthogonal test (Table 4) revealed the order of factor influence on emulsion stability as: *β*-CD concentration (A) > pH (B) > oil-to-water ratio (C). The optimal preparation conditions were determined to be A_2_B_2_C_3_, corresponding to 3 % *β*-CD, pH 7.5, and a 2:8 oil-to-water ratio. Validation of this optimal combination yielded an emulsion with a particle size of 74.19 nm and 97.73 % emulsification activity. Analysis of variance (Table 5) showed that *β*-CD concentration had an extremely significant effect on the comprehensive index (*p* < 0.01), pH had a significant effect (*p* < 0.05), and the oil-to-water ratio had no significant effect.

**Table 5:** Analysis of variance table.

Source of variance	Sum of squares	Freedom	Mean square	F value	P value
A	0.82	2	0.41	1,112.47	0.00**
B	0.02	2	0.01	23.93	0.04*
C	0.01	2	0.00	6.55	0.13
Error	0.00	2	0.00		

*and **mean significant difference at 0.05 and 0.01 level, respectively.

Although the oil-to-water ratio is a recognized factor governing emulsion stability, it did not exhibit a statistically significant effect in this orthogonal experiment (*p* > 0.05, Table 5). This may be due to the highly efficient interfacial particle coverage already achieved under the optimal conditions of 3 % *β*-CD and pH 7.5. Consequently, across the tested range of oil-to-water ratios (*φ* = 70–80 %), the system demonstrated a robust capacity to form stable Pickering emulsions. This indicates the existence of a ‘plateau region’ wherein the stabilizing effect of *β*-CD particles is less sensitive to moderate variations in the oil phase proportion, provided the emulsifier concentration and interfacial conditions are optimal.

### 3.3 Effect of fat replacement on surimi gel quality

#### 3.3.1 TPA

The impact of PPEO Pickering emulsion fat substitution on surimi gel texture was evaluated by TPA (Table 6). The results indicated significant differences in hardness and chewiness across substitution levels, with no significant effects on springiness or cohesiveness. Before F-T, hardness progressively decreased with higher substitution levels, showing significant differences from the control (*p* < 0.05). Following three F-T cycles, a significant reduction in hardness was observed in the control group (*p* < 0.05), likely due to ice crystal formation from free water, leading to cellular damage, protein structural changes, and quality loss [2]. Notably, while hardness in the emulsion groups also changed significantly after F-T, the extent of change was substantially smaller. This stabilization mechanism may involve the emulsion limiting ice crystal growth, thus reducing physical damage to proteins, minimizing solute concentration shifts and radical generation associated with large ice crystals, and retarding myofibrillar protein oxidation and aggregation [31], 32]. For chewiness, decreases were noted at 0 % and 25 % substitution after F-T, whereas a non-significant increasing trend occurred at higher substitution levels (*p* > 0.05). Collectively, although F-T cycling altered texture parameters, the changes were significantly less severe in emulsion-containing gels than in the control. This finding confirms that substituting fat with PPEO Pickering emulsion effectively suppresses texture changes and improves the F-T stability of surimi gel.

**Table 6:** Effect of PPEO Pickering emulsion replacement of fat on the texture of surimi gel.

Group	Emulsion substitution ratio (%)	Hardness (g)	Chewiness	Springiness (mm)	Cohesiveness
Before F-T cycles	0	2,995.95 ± 41.82aA	2,302.97 ± 19.15aA	0.86 ± 0.01aA	0.76 ± 0.01aA
	25	2,638.29 ± 38.68bA	2065.25 ± 39.79bA	0.85 ± 0.03aA	0.76 ± 0.01aA
	50	2,476.31 ± 31.68cA	1,884.15 ± 35.91cA	0.86 ± 0.01aA	0.75 ± 0.02aA
	75	2,169.53 ± 44.78dA	1,709.76 ± 41.09eA	0.86 ± 0.02aA	0.75 ± 0.01aA
	100	2,044.36 ± 38.27eA	1,798.16 ± 46.77dA	0.84 ± 0.02aA	0.73 ± 0.02bA
After F-T cycles	0	2,709.25 ± 16.15aB	1,850.63 ± 35.40abB	0.84 ± 0.03aA	0.75 ± 0.01aA
	25	2,623.44 ± 67.47aA	1,813.05 ± 58.20cB	0.84 ± 0.01aA	0.75 ± 0.01aA
	50	2,440.41 ± 76.08bA	1,890.28 ± 34.60aA	0.83 ± 0.02aA	0.74 ± 0.02aA
	75	2,159.38 ± 33.52cA	1,710.71 ± 55.59dA	0.84 ± 0.01aA	0.74 ± 0.01aA
	100	1,989.90 ± 122.01dA	1,809.14 ± 16.9 cA	0.85 ± 0.01aA	0.74 ± 0.01aA

Different lowercase letters indicate significant differences between groups (*p* < 0.05); different uppercase letters indicate significant differences within groups (*p* < 0.05). The same as following.

#### 3.3.2 TBARS value

**Figure 2:**
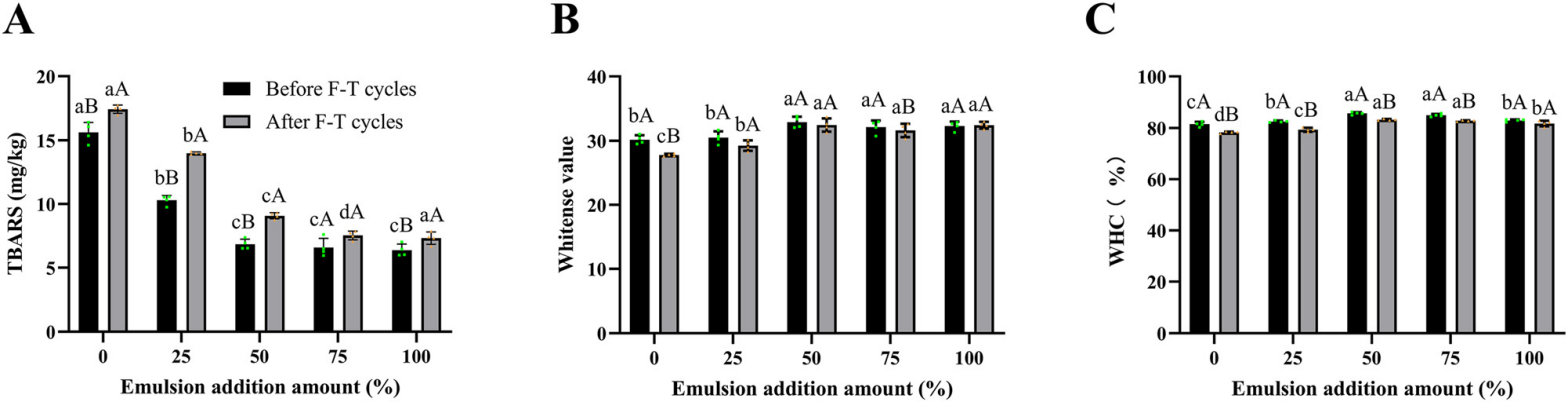
Effect of PPEO pickering emulsion replacement of fat on the TBARS value (**A**), whitense value (**B**), WHC (**C**) of surimi gel. The above values are expressed as mean ± SD (*n* = 4). Different lowercase letters indicate significant differences between groups (*p* < 0.05); different uppercase letters indicate significant differences within groups (*p* < 0.05).

The TBARS assay measures malondialdehyde, a secondary product of lipid oxidation, providing an indicator of the extent of lipid oxidation and rancidity. As shown in Figure 2A, substituting fat with the PPEO Pickering emulsion significantly lowered the TBARS value of surimi gel (*p* < 0.05), demonstrating its efficacy in inhibiting lipid oxidation. This effect is likely due to the phenolic compounds in PPEO, which act as radical scavengers by donating active hydrogen, thereby terminating oxidation chain reactions. Our findings align with previous studies on fat replacement using Pickering emulsions. For instance, Yang et al. [33] observed reduced TBARS values in sausages where pig back fat was replaced by a high internal phase Pickering emulsion, attributing the effect to a dense interfacial layer that acts as a physical oxygen barrier and provides surface antioxidant activity. Similarly, Li et al. [34] reported that a tea polyphenol-containing linseed oil double emulsion effectively lowered TBARS values in pork batter, linking the outcome to the termination of radical chain reactions by polyphenols. Following three F-T cycles, TBARS values increased across all groups compared to pre-F-T levels (Figure 2A). This rise may be attributed to structural degradation during F-T, including reduced gel strength, juice loss, and weakened emulsion-surimi integration. These changes potentially increase porosity and the contact area between lipids and pro-oxidants, accelerating oxidation [35].

#### 3.3.3 Whiteness

Whiteness, a key visual attribute influencing consumer preference, is an important quality index for surimi products. The effect of PPEO Pickering emulsion substitution on gel whiteness is shown in Figure 2B. Whiteness increased progressively with higher emulsion substitution levels. This enhancement may be attributed to the larger total surface area provided by the finely dispersed emulsion droplets, which increases light reflection (manifested as a higher L* value, indicative of lightness). This finding aligns with Wang et al. [36], who reported that an octenyl succinate starch-based Pickering emulsion similarly improved the whiteness of fish protein gels as a fat substitute. Specifically, at a 50 % substitution level, the whiteness before F-T was significantly higher than that of the control and the 25 % group, and marginally higher than the 75 % and 100 % groups. Furthermore, the whiteness of emulsion-containing gels remained largely stable after three F-T cycles, whereas the control group exhibited a significant decrease. This decline in the control likely resulted from F-T induced damage to the gel network-including ice crystal growth, moisture migration, and protein denaturation – which deteriorates optical properties [37]. Therefore, incorporating the PPEO Pickering emulsion not only improved initial whiteness but also effectively inhibited its reduction during F-T cycling, thereby enhancing F-T stability, with the 50 % substitution level demonstrating the optimal overall effect.

#### 3.3.4 WHC

WHC is crucial for the texture and juiciness of surimi products, and fat contributes to this property [38]. The effect of PPEO Pickering emulsion substitution on WHC is presented in Figure 2C. WHC initially increased and then decreased with higher substitution levels, peaking at a 50 % substitution ratio. This optimal performance likely stems from a synergistic interaction between the emulsion droplets and surimi proteins at this specific ratio, promoting protein-protein interactions and the formation of a stable, elastic gel network with enhanced water-binding capacity during cooking [39]. Conversely, excessive emulsion (beyond 50 %) may hinder the formation of a cohesive interfacial structure, leading to protein aggregation, compromised emulsification stability, and consequently, reduced WHC [35]. Following three F-T cycles, WHC declined across all groups. While the control WHC decreased from 81.51 % to 78.30 %, the values for emulsion-substituted gels remained significantly higher, ranging from 79.35 % to 83.25 %. Notably, the gel with 50 % substitution retained the highest WHC after F-T. These results demonstrate that partial fat replacement with PPEO Pickering emulsion, particularly at a 50 % ratio, not only improves the WHC of surimi gel but also enhances its retention during F-T stress, thereby boosting F-T stability.

#### 3.3.5 Microstructure

**Figure 3:**
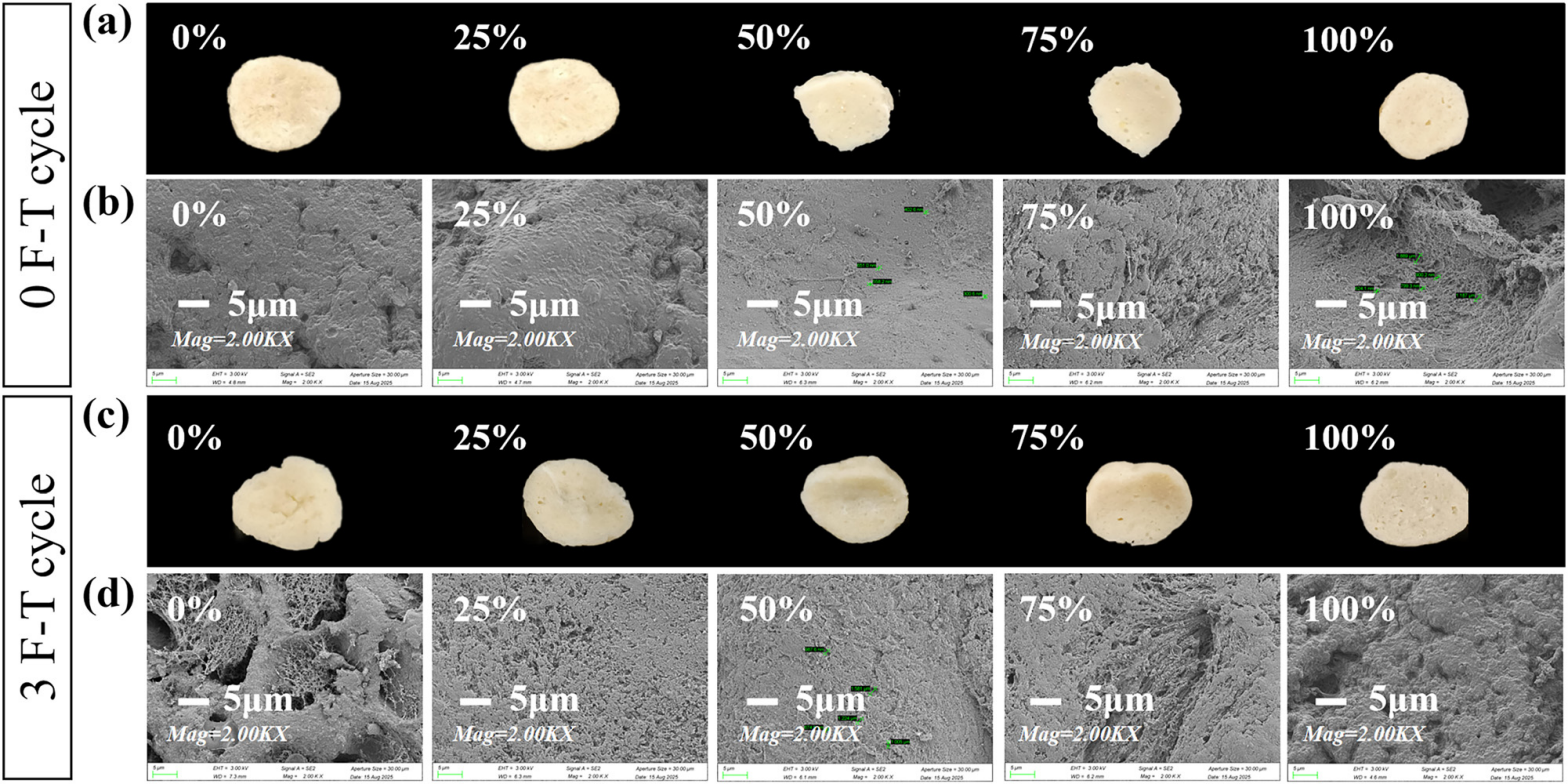
Effect of PPEO pickering emulsion as a fat substitute on the microstructure of surimi gel before (0 F-T cycle) and after (3 F-T cycle) F-T cycles. (a,c) cross-sectional morphology; (b,d) microstructural networks.

The gel structure of surimi, governed by myofibrillar protein aggregation and interactions [40], was visualized using SEM. As revealed in Figure 3, F-T cycling rendered the control gel structure loose and highly porous, a likely consequence of ice crystal expansion damaging the protein network-consistent with its decreased WHC. Conversely, gels containing PPEO Pickering emulsion displayed progressively smoother surfaces and a denser, sheet-like matrix. Optimal structural uniformity and compactness were observed at the 50 % replacement level. This enhancement may be driven by phenolic hydroxyl groups in PPEO-derived polyphenols, which can induce protein cross-linking, leading to a reinforced, intricate gel network [41]. Notably, at higher replacement levels (75%–100 %), the gel structure reverted to containing large, irregular cavities (Figure 3), potentially because excessive emulsion interferes with critical intermolecular forces, ultimately deteriorating the gel matrix [42].

The underlying mechanism entails a dual contribution from the PPEO Pickering emulsion. Physically, the submicron droplets serve as active fillers that reinforce the continuous protein phase. Molecularly, polyphenolic components at the oil-water interface may interact with myofibrillar proteins through hydrogen bonding and hydrophobic effects, fostering beneficial cross-linking that increases network elasticity and strength. During heat-induced gelation, these droplets become incorporated into the evolving protein matrix, yielding a cohesive composite structure. This synergistic integration results in a gel network with superior resistance to the mechanical and osmotic stresses imposed by F-T cycles, effectively preserving both textural and water-holding properties.

## 4 Conclusions

This study demonstrated that replacing 50 % of fat with a *β*-CD-stabilized PPEO Pickering emulsion effectively inhibited hardness loss in surimi gel after F-T cycles, alleviated ice crystal damage to the protein network, reduced color change and juice loss, improved antioxidant capacity, and strengthened the gel network, thereby enhancing F-T stability. This provides a theoretical reference for applying Pickering emulsions in developing low-fat surimi products.

Future studies should elucidate the mechanisms by which PPEO emulsion reduces ice crystal size and delays myofibrillar protein oxidation and denaturation. Advanced techniques like large amplitude oscillatory shear and oral processing-inspired tribology could further characterize the viscoelastic, fracture, and lubrication properties of the composite gel, bridging instrumental measurements and sensory perception.
